# Influence of smoking and obesity on post-COVID-19 sequelae and risk of hospitalization

**DOI:** 10.3389/fmed.2024.1499239

**Published:** 2024-12-05

**Authors:** Daniel Fernández-Pedruelo, Raúl Juárez-Vela, Regina Ruiz de Viñaspre-Hernández, Javier Alonso-Alonso, José Maríal Criado-Gutiérrez, Consuelo Sancho-Sánchez

**Affiliations:** ^1^Doctoral Program in Health, Disability, Dependency, and Well-being, Faculty of Medicine, University of Salamanca, Salamanca, Spain; ^2^Deparment of Nursing, Faculty of Health Sciences, University of La Rioja, Logroño, Spain; ^3^Deparment of Nursing, Faculty of Health Sciences, University of La Rioja, Logroño, Spain; ^4^Deparment of Psychiatry, Faculty of Valladolid, Valladolid, Spain; ^5^Department of Physiology and Pharmacology, Faculty of Medicine, University of Salamanca, Salamanca, Spain; ^6^Department of Physiology and Pharmacology, Faculty of Medicine, University of Salamanca, Salamanca, Spain

**Keywords:** COVID-19, smoking, obesity, sequelae, hospitalization

## Abstract

**Introduction:**

The COVID-19 pandemic, caused by the SARS-CoV-2 virus, has significantly impacted the global healthcare system, with particularly harmful effects on the human respiratory system. Beyond the acute symptoms, there is growing concern about persistent symptoms that last for weeks or months after the initial infection, known as long COVID syndrome. This study focuses on investigating the relationship between smoking, obesity, and the presence of post-COVID-19 sequelae, as well as their influence on the risk of hospitalization.

**Materials and methods:**

An observational and retrospective study was conducted using medical records of patients diagnosed with COVID-19 in Castilla y León, Spain, between November 1 and 30, 2020. The patients were divided into three groups: smoking (current and former), obesity/overweight, and control group. Various variables were analyzed, including age, sex, and the presence of post-COVID-19 sequelae, chronic pathologies, cardiovascular diseases, psychological conditions, and hospitalization. Descriptive statistics and Odds Ratio analysis were used for comparisons.

**Results:**

The results revealed that obesity was significantly associated with a higher risk of post-COVID-19 sequelae, particularly memory disorders and neurological, mental, or psychological symptoms. In contrast, smoking was correlated with an increase in memory problems but did not show a direct influence on post-COVID-19 sequelae or hospitalization. Additionally, women were found to have a higher prevalence of obesity in the studied population.

**Conclusion:**

This study provides evidence that obesity increases the risk of post-COVID-19 sequelae, especially in terms of memory disorders and neuropsychological symptoms. On the other hand, smoking is related to memory problems. Regarding cardiovascular pathologies, there was not enough statistical evidence for analysis, while for hospitalization, it was determined that smoking and obesity do not have a direct influence on these post-COVID consequences.

## 1 Introduction

The COVID-19 pandemic, caused by the SARS-CoV-2 virus, has had an unprecedented impact on global health and has posed numerous challenges for the medical and scientific community worldwide. While the acute symptoms of the disease, such as fever, cough, and difficulty breathing, have been widely documented, it has become increasingly evident that the virus’s impact is not limited to the acute period of infection. COVID-19, caused by the SARS-CoV-2 virus, primarily affects the human respiratory system, with symptoms manifesting within one to 2 weeks (1). However, studies show a growing trend of patients experiencing post-COVID symptoms ranging from 30 days to 12 weeks after diagnosis, known as long COVID syndrome (2–4).

The scientific literature has documented the presence of persistent symptoms that last for weeks or even months after the initial infection, giving rise to what is known as long COVID syndrome or “Long COVID.” Long COVID symptoms vary widely and include extreme fatigue, memory loss, difficulty concentrating, headaches, and problems with smell and taste, among others (5). Also, Long COVID symptoms may persist for more than 2 years (6).

There are mechanisms that are being studied as potential factors behind the development of long COVID including the persistence of SARS-CoV-2 RNA in reservoir cells (7, 8) and the potential role of autoantibodies (9). However, researchers are studying preventive measures that can reduce the risk of long COVID development (10–12)

On the other hand, obesity is another health condition identified as a risk factor for COVID-19 (13). Obese patients may experience a greater need for mechanical ventilation, increasing their risk of hospitalization. Additionally, obesity has been linked to a higher prevalence of chronic conditions such as diabetes and hypertension, which can also increase the severity of COVID-19 (14).

However, despite the growing concern about the impact of smoking and obesity on COVID-19 patients, evidence on these associations is still inconclusive. Some studies have found significant links, while others have reported contradictory results (15, 16).

### 1.1 Objective

The primary objective of this study is to investigate the influence of smoking and obesity on the occurrence of post-COVID-19 sequelae and the risk of hospitalization. To achieve this, we conducted a detailed analysis of a group of patients diagnosed with COVID-19 during a specific period, evaluating multiple variables and using descriptive statistics and Odds Ratios to gain a clearer understanding of these associations.

### 1.2 Hypothesis

Therefore, as research on Long COVID progresses, understanding the risk factors that may contribute to the occurrence and severity of these sequelae has become essential. We hypothesize that previous health factors, such as smoking and obesity, increase the probability of suffering persistent symptoms after the acute phase of COVID-19. Smoking is a known risk factor for respiratory diseases and has been a concern in relation to COVID-19 infection due to its detrimental effects on lung function and the respiratory system (17). Given the virus’s primary impact on the lungs, there is a hypothesis that smoking could increase the risk of severe complications in COVID-19 patients (18). Obesity appears as a risk factor, since prolonged metabolic and inflammatory dysfunction could delay recovery and exacerbate prolonged symptoms, such as fatigue, muscle pain and respiratory distress, thus increasing vulnerability to long-lasting sequelae (19).

*H1*: Smoking increases the risk of having Long COVID sequalae symptoms.

*H2*: Obesity increases the risk of having Long COVID sequalae symptoms.

## 2 Materials and methods

### 2.1 Investigation design

An observational and retrospective study was conducted to investigate the influence of smoking and obesity on post-COVID-19 sequelae and the risk of hospitalization. This research design allowed for the analysis of previously collected data and the generation of comparative results between different groups of patients.

### 2.2 Study population

The study population consisted of patients diagnosed with COVID-19 through polymerase chain reaction (PCR) tests conducted by professionals from the public health system of Castilla y León between November 1, 2020, and November 30, 2020, and recorded in the “MEDORA” electronic health record system of the public health system “SACYL,” who follows WHO guidelines to identify symptoms. These dates were selected to ensure that the data were representative of a specific period of the pandemic. They were 56.32% of women in the smoking group (401 male, 517 female), 59.92% in the obesity group (515 male, 770 female), and 54.30% in the control group (6,497 male, 7,719 female). For detailed data about the study population, check Table 1.

**Table 1:** Distribution of patients according to characteristics and post COVID-19 sequelae.

	Smoking (current and former)	Obesity and overweight	Control group
Male sex	401	515	6.497
Female sex	517	770	7.719
Age	42.65 (22–64)	34.33 (19–52)	36.37 (16–68)
Chronic conditions	0	3	11
Cardiovascular diseases	0	0	1
Long COVID sequelae	12	44	256
Memory disorders and loss	9	14	59
Hospitalization	0	3	36

### 2.3 Inclusion criteria

1. Hospitalization: The risk of hospitalization within twelve months following a positive PCR diagnosis of COVID-19 was recorded (Continuous variable).
2. Belong to one of the three defined study groups: patients with a history of smoking (current or former), patients with obesity or overweight (with a body mass index [BMI] over 25), and a third control group composed of individuals who have never smoked and are not overweight or obese.
3. For the control group, individuals who did not meet the characteristics of the study groups and did not have chronic conditions such as hypertension (HTN), diabetes, or dyslipidemia were included to ensure the homogeneity of the results.

### 2.4 Exclusion criteria

Patients with a history of chronic conditions such as hypertension (HTN), diabetes, and dyslipidemia before contracting COVID-19 were excluded. For the control group, individuals with current or past issues of smoking or obesity were also excluded.

### 2.5 Sample size

**Figure 1:**
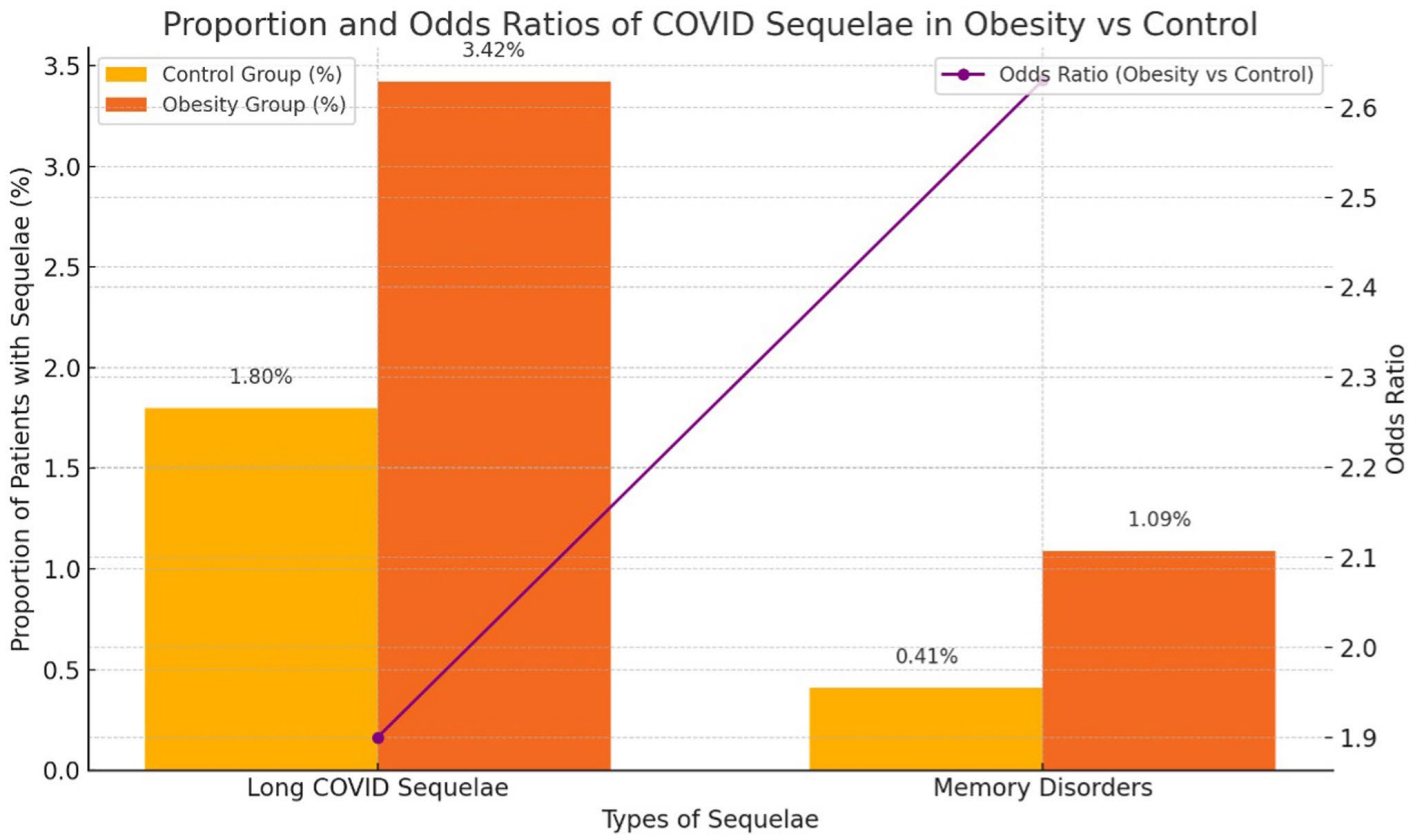
Proportion and Odds Ratios of COVID Sequelae in Obesity vs Control.

Initially, the database contained information on 27,184 patients. However, following the previously mentioned inclusion and exclusion criteria, the study sample was reduced to a total of 16,434 patients. The 10,750 excluded patients did not meet the defined characteristics and are detailed in the Flowchart of Results (Figure 1) presented.

Table 1 shows the characteristics of the study groups according to age, sex, and the number of patients who presented any type of post-COVID-19 sequelae, classified by each group and the control group. Memory disorders and memory loss have been analyzed independently of other post-COVID sequelae because only physical sequelae were considered within the group, while these are neurological, mental, or psychological in nature, and therefore have been analyzed separately.

### 2.6 Sample characterization

To characterize the sample, the following exact variables were analyzed:

- *Age:* The average age (Discrete variable) of the patients in each group was calculated, as well as the age range (minimum and maximum). The mean age in the sample was 48,8 years, with a standard deviation of 24,6 years.
- *Sex:* The gender proportion in each group was determined (Binary variable, 0 = male, 1 = female). They were 56.32% of women in the smoking group, 59.92% in the obesity group, and 54.30% in the control group.
- *Chronic conditions:* The presence or absence of chronic conditions, including HTN, dyslipidemia, and diabetes, was recorded (Binary variable, 0 = Absence, 1 = Presence).
- *Cardiovascular diseases:* The presence or absence of cardiovascular diseases, such as heart attack and heart failure, was recorded (Binary variable, 0 = Absence, 1 = Presence).
- *Long COVID sequelae:* The presence of sequelae such as headaches, expectoration, myalgias, fatigue, and taste and smell alterations was analyzed (Binary variable, 0 = Absence, 1 = Presence).
- *Memory disorders and memory loss:* The incidence of memory disorders and memory loss was evaluated (Binary variable, 0 = Absence, 1 = Presence).
- *Hospitalization*: The hospitalization risk of patients was recorded (Continuous variable).

### 2.7 Statistical analysis

Descriptive statistics were used to characterize the sample, and Odds Ratios (OR) were calculated to analyze the relationships between the independent variables (smoking and obesity) and the dependent variables (post-COVID-19 sequelae, chronic conditions, cardiovascular diseases, and hospitalization risk). A 95% confidence level was considered for these analyses, corresponding to a 5% significance level *(p = 0.05).* The statistical software SPSS V25.0 (New York, United States) was used for the analyses.

#### 2.7.1 Ethical considerations

This study was approved by the Bioethical Committee of the University of Salamanca (registration number 734). The study was conducted in accordance with the principles of the Declaration of Helsinki and the recommendations of good clinical practice. For reporting, the Strengthening the Reporting of Observational Studies in Epidemiology (STROBE) guidelines were followed.

## 3 Results

In this section, detailed results of our study are presented, focused on investigating the intricate relationships between obesity, smoking, and post-COVID-19 sequelae in recovered patients. These findings offer a profound understanding of how these variables influence population health following COVID-19 infection.

### 3.1 Patient characterization

Initially, we characterized the participants selected for the study. The analysis revealed significant differences in the average ages of the groups: the smoking group had a mean age of 42.65 years, the obesity group showed a mean age of 34.33 years, and the control group had a mean age of 36.37 years. Additionally, we observed a predominance of females in all groups, with 56.32% of women in the smoking group, 59.92% in the obesity group, and 54.30% in the control group.

### 3.2 Sequelae and pathologies post-COVID-19

Next, we examined the sequelae and pathologies following COVID-19 infection. The most common sequelae were “Long COVID” symptoms such as headache, cough, myalgia, fatigue, and changes in taste and smell. These manifested in 3.42% of patients with obesity, 1.8% of the control group, and 1.34% of smokers, indicating a higher incidence among patients with obesity.

Memory disorders occurred in 1.1% of patients with obesity, 0.98% of smokers, and 0.42% of the control group, showing a higher incidence in the obesity and smoking groups.

For chronic conditions and hospitalization risk, the smoking group showed no cases, while patients with obesity had a 0.23% incidence in both categories. The control group had a 0.08% incidence in chronic conditions and 0.25% in hospitalization risk. Lastly, a single cardiovascular case was noted in the control group, with no cases in the experimental groups.

### 3.3 Simple and relative risk factors

Tables 2, 3 illustrate the simple risk and the odds ratio of the study groups to experience any of the analyzed sequelae.

**Table 2:** Simple risk results.

Variable	Chronic conditions (hypertension, dyslipidemia, diabetes)	Long Covid sequelae (headache, cough, myalgia, fatigue, taste and smell alterations)	Memory disorders and loss	Hospitalization
Healthy	0.08%	1.80%	0.41%	0.25%
Smoking (current and former)	–	1.31%	0.98%	–
Obesity and overweight	0.23%	3.42%	1.09%	0.23%
Between experimental groups	–	2.54%	1.04%	–

**Table 3:** Odds ratio results.

Variable	Chronic conditions (hypertension, dyslipidemia, diabetes)	Long Covid Sequelae (headache, cough, myalgia, fatigue, taste and smell alterations)	Memory disorders and loss	Hospitalization
Obesity and overweight / healthy	3.01	1.90	2.63	0.92
Smoking / healthy	–	0.73	2.36	–
Obesity and overweight / smoking	–	2.62	1.11	–
RRR: odds ratio				

### 3.4 CHI-SQUARE test results

Below are the results obtained using the CHI-SQUARE methodology, which allows for evaluating the dependency relationship between the risk of experiencing sequelae or hospitalization risk during the post-COVID period and the obesity and smoking pathologies of the patients (Table 4). A specific confidence level was used to define the significance of the relationship *(p = 0.05)*.

**Table 4:** Results of χ^2^ analysis for the level of risk of previous chronic conditions.

Variable	Chronic conditions (hypertension, dyslipidemia, diabetes)			Long Covid sequelae (headache, cough, myalgia, fatigue, taste and smell alterations)			Memory disorders and loss			Hospitalization		
	Est. test	*χ* ^2^	*p*-value	Est. test	*χ* ^2^	*p*-value	Est. test	*χ* ^2^	*p*-value	Est. test	*χ* ^2^	*p*-value
Smoking (current and former)	–	3.8	–	1.2	3.8	0.27	6.18	3.8	0.01	–	3.8	–
Obesity and Overweight	3.17	3.8	0.07	16.42	3.8	0.00	11.46	3.8	0.00	0.02	3.8	0.89
Between experimental groups	–	3.8	–	9.69	3.8	0.00	0.06	3.8	0.80	–	3.8	–

### 3.5 Relationships between variables of interest and their implications in post-COVID-19 health

#### 3.5.1 Chronic conditions and their association with obesity

In the first analysis (Table 5), we investigated the potential association between the presence of chronic conditions (Hypertension, Dyslipidemia, and Diabetes) and obesity status in a representative sample of the post-COVID-19 population. The results of the CHI-SQUARE test (X^2) indicated a lack of significant association between these two variables (Test = 3.17 < X^2 = 3.84, *p < 0.05*), suggesting that obesity may not be directly related to the presence of these chronic conditions in COVID-19 recovered patients.

**Table 5:** Chronic conditions in relation to obesity.

	With chronic conditions	Without chronic conditions	Total
Obesity and overweight	3	1,285	1,288
None	11	14,220	14,231
Total	14	15,505	15,519

#### 3.5.2 Post-COVID-19 sequelae and their relationship with smoking

Next (Table 6), we explored the relationship between the presence of post-COVID-19 sequelae (Headache, Cough, Myalgias, Fatigue, Taste and smell alterations) and smoking history (either current or former). The results revealed that there is no significant association between these two variables (Test = 1.20 < X^2 = 3.84, *p < 0.05*). This indicates that smoking may not directly be related to the occurrence of post-COVID-19 sequelae in the studied patients.

**Table 6:** Long Covid sequelae in relation to obesity and smoking

	With long Covid sequelae	No long Covid sequelae	Total
Smoking	12	906	918
Obesity and overweight	44	1,241	1,285
None	256	13,975	14,231
Total	268	14,881	16,434

#### 3.5.3 Post-COVID-19 sequelae and their relationship with obesity

Later (Table 6), we focused on the association between obesity and the presence of post-COVID-19 sequelae. The results showed a significant relationship between these two variables (Test = 16.42 > X^2 = 3.84, *p < 0.05*), suggesting that patients with obesity may be more likely to experience post-COVID-19 sequelae compared to those without obesity.

#### 3.5.4 Interaction between obesity and smoking in post-COVID-19 sequelae

Afterwards (Table 6), we evaluated the potential interaction between obesity and smoking in the occurrence of post-COVID-19 sequelae. The results revealed a significant association between these variables (Test = 9.69 > X^2 = 3.84, *p < 0.05*), indicating that patients with obesity and a history of smoking may have a significantly higher risk of developing post-COVID-19 sequelae compared to other groups.

#### 3.5.5 Memory disorder and loss in post-COVID-19 patients in relation to smoking

In the fifth analysis (Table 7), we examined the presence of memory disorders in COVID-19 recovered patients in relation to their smoking history. The results demonstrated a significant relationship between these variables (Test = 6.18 > X^2 = 3.84, *p < 0.05*), suggesting that patients with a history of smoking may have a higher risk of experiencing memory disorders after COVID-19 infection.

**Table 7:** Memory disorder and loss in relation to obesity and smoking.

	With memory disorder and loss	Without memory disorder and loss	Total
Smoking	9	909	918
Obesity and overweight	14	1,271	1,285
None	59	14,172	14,231

#### 3.5.6 Memory disorder and loss in post-COVID-19 patients in relation to obesity

In the next analysis (Table 7), we explored the association between obesity and memory disorders in post-COVID-19 patients. The results indicated a significant relationship between these variables (Test = 11.46 > X^2 = 3.84, *p < 0.05*), suggesting that obesity may be associated with a higher risk of memory disorders in COVID-19 recovered patients.

#### 3.5.7 Interaction between obesity, smoking, and memory disorders

To follow (Table 7), we investigated the potential interaction between obesity, smoking, and memory disorders in post-COVID-19 patients. The results did not show a significant association between these variables (Test = 0.06 < X^2 = 3.84, *p < 0.05*), indicating that these variables may be independent of each other in the studied population.

#### 3.5.8 Interaction between obesity and hospitalization risk

Finally (Table 8), we investigated the potential interaction between obesity and hospitalization risk in post-COVID-19 patients. The results did not show a significant association between these variables (Test = 0.02 < X^2 = 3.84, *p < 0.05*), indicating that these variables may be independent of each other in the studied population.

#### 3.5.9 Comparative analysis of obesity and smoking with Long COVID sequelae

**Table 8:** Hospitalization based on obesity.

	With hospitalization	Without hospitalization	Total
Obesity and overweight	3	1,282	1,285
None	36	14,195	14,231
Total	39	15,477	15,516

On the other hand, based on the test statistic to determine the relationship between obesity and smoking with Long COVID sequelae, it was established that smoking does not represent a risk factor that increases the likelihood of experiencing this type of sequelae, while obesity does have a direct relationship with the increased likelihood of incidence of these consequences in patients affected by it. When conducting a comparative analysis between patients with smoking and obesity, it was determined that overweight individuals have a higher percentage of risk than tobacco consumers to suffer from Long COVID sequelae.

Regarding memory disorders and loss, the test statistics were higher than the X^2, both for patients with smoking and those with obesity. Therefore, it can be defined that the risk of these sequelae occurring increases in people who smoke and/or have overweight or obesity issues. Finally, regarding hospitalization risk, it was determined that it was not influenced by any of these chronic conditions.

Overall, our findings underscore the importance of understanding the complex interactions between obesity, smoking, and post-COVID-19 sequelae in the health of recovered patients. These results may have significant implications for healthcare and the planning of prevention and treatment strategies in the post-COVID-19 pandemic era.

To confirm the results obtained in the X^2 test, odds ratios were determined to establish the probability risk relationship of experiencing some of the analyzed post-COVID sequelae among each established population group. Each disease was evaluated separately.

From these results, the following findings can be established:

- Patients with obesity are 3.01 times more likely to suffer from chronic conditions (Hypertension, Dyslipidemia, Diabetes) post-COVID than those without such issues prior to the coronavirus diagnosis.
- Similarly, with Long COVID sequelae (headache, cough, myalgias, fatigue, taste and smell alterations), it was determined that individuals with obesity have a 1.92 times higher probability of experiencing these conditions in the post-COVID period.
- Regarding memory loss and disorders, it was determined that patients with obesity and smoking have a higher risk index than those without these pre-existing conditions, resulting in odds ratios of 2.38 and 2.65, respectively.

On the other hand, it was determined that patients with obesity have a 1.11 times higher probability of experiencing memory disorders and loss compared to those with a history of smoking, a difference that can be considered relatively insignificant.

## 4 Discussion

This study has focused on analyzing the possible relationship between the risk of developing chronic, cardiovascular, and psychological sequelae, as well as the risk of hospitalization in the post-COVID period, with smoking and obesity. The results cover a one-year period and include patients of all ages. Adding interest to this research is the fact that it is the first analysis of its kind conducted in the Castilla y León region. According to the results obtained, the following analyses are presented.

Based on the results obtained, it was determined that the three groups consist mostly of women, with 59.92% of the female population being obese patients, 56.32% being smokers, and 54.30% being categorized as healthy. Of the total selected patients, it was determined that 1.90% presented cardiovascular sequelae, which is similar to the findings presented by studies by other authors, who established that the incidence rate of this type of sequelae ranges from 1.82 to 2.42%, translating to 179 to 236 cases annually per 100,000 inhabitants (20–23).

On the other hand, the results show that the incidence rate of Long COVID sequelae (headache, cough, myalgias, fatigue, taste and smell alterations) was 1.31%, which differs from the results presented by Jiménez et al. (17), who established an incidence rate of 7.63% of this type of post-COVID disease in smokers. Additionally, this study determined that smokers do not have a higher risk of presenting Long COVID sequelae than those who do not smoke, which also contradicts the results of another study, where it was determined that the smoking population had a 25.6% higher probability of suffering from any of these post-COVID diseases.

*H1*: Smoking increases the risk of having Long COVID sequalae symptoms.

The hypothesis is rejected.

Regarding Long COVID sequelae in patients with obesity, an incidence rate of 3.42% was determined, which is below the probability risk range defined in other research, where incidence rates between 10.5 and 33.3% were defined, resulting in a risk level three to ten times lower than that defined by other research (24, 25). When comparing the risk percentage of obese patients to those without this condition, this study determined an incidence 1.92% higher, which is similar to the results shown by other studies that establish odds ratios in a range between 1.58 and 2.0 (26, 27).

*H2*: Obesity increases the risk of having Long COVID sequalae symptoms.

The hypothesis is accepted.

For the case of sequelae related to memory disorders and loss, a risk percentage of 0.50% of the total study population was determined. These results are lower than those presented by the study of Soraas (28), which established an incidence rate of 4%, with memory loss being the most common sequelae among the psychological sequelae of Long COVID. Unfortunately, there are no previous studies evaluating the influence of obesity and smoking on the risk of memory disorders post-COVID-19, which would allow for a comparison with the results obtained in the present study.

Finally, during the present study, it was determined that obesity does not increase the risk level of hospitalization in COVID-19 patients. In fact, an odds ratio of 0.92% was determined compared to the population without obesity, which can be considered a 1:1 relationship. This contradicts the results presented by Rodríguez et al. (29), where it was determined that obese individuals had a 33% higher likelihood of hospitalization compared to those without overweight issues. However, it is important to note that our study specifically examines the risk of hospitalization within 12 months following a COVID-19 diagnosis, a timeframe not addressed in Rodríguez et al.’s analysis. To our knowledge, no previous research has determined the increased risk of hospitalization in the 12 months post-diagnosis for COVID-19 patients, highlighting the unique contribution of our findings.

In this scientific article, a thorough analysis of the relationship between smoking, obesity, and post-COVID-19 sequelae has been conducted in the Castilla y León region. This study represents a valuable contribution to understanding the long-term effects of the disease, considering significant risk factors such as tobacco consumption and elevated body mass index. Through the evaluation of a large group of patients over a one-year period, results have been obtained that provide essential information for medical care and future research. A significant prevalence of cardiovascular sequelae and Long COVID symptoms has been observed in the studied population. These findings align with previous research, although notable differences in the incidence of some sequelae in relation to smoking and obesity have been recorded.

Regarding the relationship between smoking and Long COVID sequelae, this study contradicts previous findings suggesting a higher risk in smokers. The results show that smoking does not seem to significantly increase the likelihood of developing these sequelae in post-COVID-19 patients. However, this finding raises additional questions about potential interactions between smoking and other risk factors. On the other hand, it has been confirmed that obesity is associated with a higher risk of experiencing Long COVID sequelae, although at a lower risk level than defined in previous research. This highlights the importance of considering body mass index as a relevant risk factor in planning long-term care strategies for COVID-19 patients.

Regarding sequelae of memory disorders and loss, a lower incidence has been identified compared to other studies, although the influence of obesity and smoking on these sequelae still requires further exploration.

Finally, concerning hospitalization, it has been demonstrated that obesity does not significantly increase the risk of hospitalization within 12 months following COVID-19 diagnosis in COVID-19 recovered patients.

## Data Availability

The original contributions presented in the study are included in the article/supplementary material, further inquiries can be directed to the corresponding author.

## References

[1] Guan W Ni Z Hu Y Liang W Ou C He J. Clinical characteristics of coronavirus disease 2019 in China. N Engl J Med. (2020) 382:1708–20. doi: 10.1056/NEJMoa2002032 32109013 PMC7092819

[2] Darley D. Dore G. Cysique L. Wilhelm K. Andresen D. (2020). High rate of persistent symptoms up to 4 months after community and hospital-managed SARS-CoV-2 infection. Res Lett Med J Aust. Available at: https://www.mja.com.au/journal/2020/high-rate-persistent-symptoms-4-months-after-community-and-hospital-managed-sars-cov-2 (accessed April 15, 2023) 10.5694/mja2.50963 PMC8014234 33657671

[3] Herrera J. Arellano E. Juárez L. Contreras R. (2020). Persistencia de síntomas en pacientes después de la enfermedad por coronavirus en un hospital de tercer nivel de Puebla, México. Med Int Méx; 36, pp. 789–793. Available at: https://www.medigraphic.com/cgi-bin/new/resumen.cgi?IDARTICULO=96464 (Accessed April 15, 2023).

[4] Darley DR Dore GJ Cysique L Wilhelm KA Andresen D Tonga K. Persistent symptoms up to four months after community and hospital-managed SARS-CoV-2 infection. Med J Aust. (2021) 214:279–80. doi: 10.5694/mja2.50963 33657671 PMC8014234

[5] Davis H McCorkell A WeiRyan H Re'emSigne L Akrami A. Characterizing long COVID in an international cohort: 7 months of symptoms and their impact. EClinicalMedicine. (2021) 38:101019. doi: 10.1016/j.eclinm.2021.101019 34308300 PMC8280690

[6] Fernandez-de-Las-Peñas C Notarte KI Macaset R Velasco JV Catahay JA Ver AT. Persistence of post-COVID symptoms in the general population two years after SARS-CoV-2 infection: a systematic review and meta-analysis. J Infect. (2023) 88:77–88. doi: 10.1016/j.jinf.2023.12.004 38101521

[7] Fernández-de-Las-Peñas C Torres-Macho J Macasaet R Velasco JV Ver AT Culasino Carandang THD. Presence of SARS-CoV-2 RNA in COVID-19 survivors with post-COVID symptoms: a systematic review of the literature. Clin Chem Lab Med. (2024) 62:1044–52. doi: 10.1515/cclm-2024-0036, PMID: 38366966

[8] Gaebler C Wang Z Lorenzi JCC Muecksch F Finkin S Tokuyama M. Evolution of antibody immunity to SARS-CoV-2. Nature. (2021) 591:639–44. doi: 10.1038/s41586-021-03207-w 33461210 PMC8221082

[9] Notarte KI Carandang THDC Velasco JV Pastrana A Ver AT Manalo GN. Autoantibodies in COVID-19 survivors with post-COVID symptoms: a systematic review. Front Immunol. (2024) 15:1428645. doi: 10.3389/fimmu.2024.1428645, PMID: 39035011 PMC11257835

[10] Fernández-de-Las-Peñas C Torres-Macho J Catahay JA Macasaet R Velasco JV Macapagal S. Is antiviral treatment at the acute phase of COVID-19 effective for decreasing the risk of long-COVID? A systematic review. Infection. (2024) 52:43–58. doi: 10.1007/s15010-023-02154-0, PMID: 38113020

[11] Notarte KI Catahay JA Velasco JV Pastrana A Ver AT Pangilinan FC. Impact of COVID-19 vaccination on the risk of developing long-COVID and on existing long-COVID symptoms: a systematic review. EClinicalMedicine. (2022) 53:101624. doi: 10.1016/j.eclinm.2022.101624, PMID: 36051247 PMC9417563

[12] Davis HE McCorkell L Vogel JM Topol EJ. Long COVID: major findings, mechanisms and recommendations. Nat Rev Microbiol. (2023) 21:133–46. doi: 10.1038/s41579-022-00846-2 36639608 PMC9839201

[13] Sattar N McInnes I McMurray J. Obesidad es un factor de riesgo de infección grave por COVID-19. Circulation. (2020) 142:4–6. doi: 10.1161/CIRCULATIONAHA.120.047659 32320270

[14] Berlin D Gulick R Martinez F. Severe Covid-19. N Engl J Med. (2020) 383:2451–60. doi: 10.1056/NEJMcp2009575 32412710

[15] Cai G Bossé Y Xiao F Kheradmand F Amos C. Tobacco smoking increases the lung gene expression of ACE2, the receptor of SARS-CoV-2. Am J Respir Crit Care Med. (2020) 201:1557–9. doi: 10.1164/rccm.202003-0693LE 32329629 PMC7301735

[16] Patanavanich R Glantz S. Smoking is associated with COVID-19 progression: a Meta-analysis. Nicotine Tob Res. (2020) 22:1653–6. doi: 10.1093/ntr/ntaa082 32399563 PMC7239135

[17] Jiménez C López D Alonso A. COVID-19 and smoking: a systematic review and Meta- analysis of the evidence. Arch Bronconeumol. (2021) 57:21–34. doi: 10.1016/j.arbres.2020.06.024 PMC7381922 34629638

[18] Fieiras C Panoso C Rosell C Franco J. Manejo de los síntomas persistentes de COVID-19 en atención primaria. Evidencia. (2020) 23:e002103–33. doi: 10.51987/evidencia.v23i4.6895

[19] Sudre CH Murray B Varsavsky T Graham MS Penfold RS Bowyer RC. Attributes and predictors of Long-COVID: analysis of COVID cases and their symptoms collected by the Covid Symptoms Study App. Nat Med. (2021) 27:626–31. doi: 10.1038/s41591-021-01292-y 33692530 PMC7611399

[20] Tormo M García J Cirera L Contreras J Martínez G Rodríguez M. Epidemiología del infarto agudo de miocardio en la Región de Murcia. Dirección General de Salud Pública: Consejería de Sanidad (2003).

[21] Marrugat J Sala J. Myocardial infarction in Girona, Spain: attack rate, mortality rate and 28-day case fatality in 1988. Regicor Study Group. J Int Epidemiol. (2008) 46:1173–9. doi: 10.1016/0895-4356(93)90116-i 8410101

[22] Carod F. Síndrome post-COVID-19: epidemiología, criterios diagnósticos y mecanismos patogénicos implicados. Rev Neurol. (2021) 72:384–96. doi: 10.33588/rn.7211.2021230 34042167

[23] Sans S Puigdefabregas A Paluzie G Monterde D Balaguer I. Increasing trends of acute myocardial infarction in Spain: the MONICA-Catalonia study. Eur Heart J. (2005) 26:505–15. doi: 10.1093/eurheartj/ehi068, PMID: 15618037

[24] Klang E Kassim G Soffer S Reich D. Severe obesity as an independent risk factor for COVID- 19 mortality in hospitalized patients younger than 50. Obesity (Silver Spring). (2020) 28:1595–9. doi: 10.1002/oby.22913 32445512 PMC7283736

[25] Palaiodimos L Kokkinidis D Arora S. Severe obesity, increasing age and male sex are independently associated with worse in-hospital outcomes. Metabolism. (2020) 108:154–262. doi: 10.1016/j.metabol.2020.154262 PMC7228874 32422233

[26] Hajifathalian K Kumar S Krisko T. Obesity is associated with worse outcomes in COVID-19. Clin Infect Dis. (2020) 69:90–112. doi: 10.1002/oby.22923 PMC7283831 32470210

[27] Lighter J Phillips M Hochman S Francois F. Obesity in patients younger than 60 years is a risk factor for COVID-19 hospital admission. Clin Infect Dis. (2020) 71:896–7. doi: 10.1093/cid/ciaa415 32271368 PMC7184372

[28] Soraas A. Problemas de memoria ocho meses después del COVID-19. Siic Salud. (2020) 4:1–4.

[29] Rodríguez R Expósito A Feria G. Obesidad y COVID-19: una díada peligrosa. An Acad Cienc Cuba. (2022) 12

